# Public Health Round-up

**DOI:** 10.2471/BLT.18.011018

**Published:** 2018-10-01

**Rohingya crisis: one year on**

Senora and son, Fahim, receiving clean water at a camp for displaced people in Cox’s Bazar, more than one year since the arrival of nearly 700 000 Rohingyas from Myanmar. During this time, the Government of Bangladesh, the World Health Organization (WHO) and health partners have helped save thousands of lives including through the prevention and containment of infectious disease outbreaks.

**Figure:**
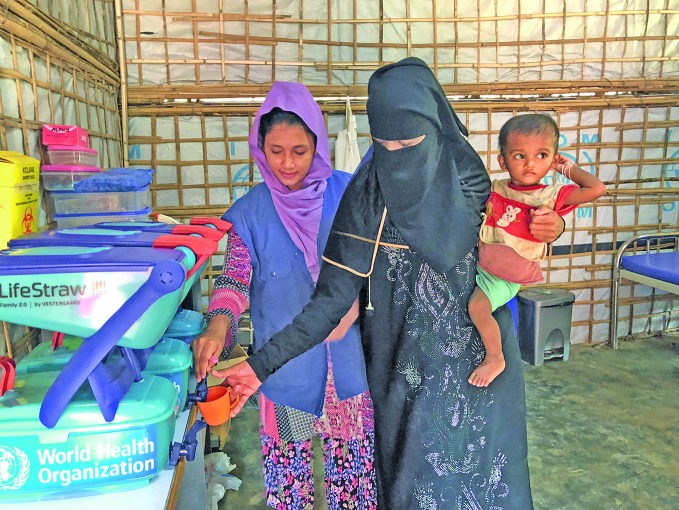
WHO/K. Marshall

## Food security

After a decade of decline, hunger is on the increase in many countries, according to *The state of food security and nutrition in the world*, a report by the Food and Agriculture Organization of the UN (FAO), the International Fund for Agricultural Development (IFAD), the World Food Program (WFP), UNICEF and the World Health Organization (WHO). The agencies estimate that the number of people in the world affected by undernourishment, or chronic food deprivation, has increased from 804 million in 2016 to nearly 821 million in 2017. 

The report found that direct and indirect climate-driven impacts have a cumulative effect, leading to increased food insecurity and malnutrition. Severe droughts in 2015–2016 directly affected many countries where the livelihood of a high proportion of the population depends on agriculture. Crop failure then contributed to the recent increase in undernourishment at the global level.

In their foreword, leaders of FAO, IFAD, WFP, UNICEF and WHO said that, “The alarming signs of increasing food insecurity and high levels of different forms of malnutrition are a clear warning that there is considerable work to be done to make sure we “leave no one behind” on the road towards achieving the SDG goals on food security and improved nutrition.” 

bit.ly/2O8xHNO

## Stimulating nutrition research

International experts will gather this month in London to generate new ideas and establish new collaborations for nutrition research. 

The meeting, *Transforming Nutrition Science for Better Health, *will be held by the Wellcome Trust and the World Health Organization (WHO) from 15-17 October 2018.

The aim is to stimulate interdisciplinary and collaborative approaches to nutrition science, inspire early career researchers and to draw public attention to the importance of research on nutrition. 

The meeting will focus on resilience and recovery of lean tissue, specifically muscle; and the influence of the microbiome, the “mini-ecosystem” of bacteria, fungi and viruses within the human body. 

Key presentations will be live streamed.

http://bit.ly/2oWZKEX

## Measles in the European Region

WHO is working closely with Member States in the WHO European Region facing measles outbreaks to implement response measures appropriate for each context, including enhanced routine and supplemental immunization, as well as heightened surveillance to quickly detect cases. More than 41 000 children and adults in the region were infected with measles in the first 6 months of 2018, with 37 deaths. 

France, Georgia, Greece, Italy, the Russian Federation, Serbia and Ukraine have each reported over 1000 infections in children and adults this year. Ukraine has been the hardest hit with over 23 000 people affected.

The total number for this period far exceeds the European Region’s 23 927 cases in 2017 and 5273 cases in 2016. Total reported deaths in the WHO European Region due to measles were 13 and 36 in 2016 in 2017 respectively.

The measles virus is highly contagious and spreads easily among people who have not been vaccinated or previously infected. 

To prevent outbreaks, at least 95% routine immunization coverage with both doses of vaccine is needed every year in every community. In addition, efforts are needed to reach the children, adolescents and adults who missed routine vaccinations in the past.

http://bit.ly/2oZyAxe

## Suicide prevention

WHO developed a guide entitled *Preventing suicide: a community engagement toolkit, *in collaboration with the Mental Health Commission of Canada.

Suicide is a serious public health problem that can be prevented with timely, evidence-based and often low-cost interventions. 

The guide outlines measures that can be taken to prevent suicide and suicide attempts, such as restrictions on over-the-counter analgesics, restricted access to pesticides or firearms, and policies to reduce the harmful use of alcohol. 

Other measures include early identification, treatment and care of people with mental health and substance use disorders, chronic pain and acute emotional distress, as well as training in the assessment and management of suicidal behaviour; responsible reporting by media; follow up of people who have attempted suicide; and provision of community support.

http://bit.ly/2OaoDrQ

**Cover photo**

Children waiting their turn for preliminary eye screening in the remote hills of Nepal.

**Figure:**
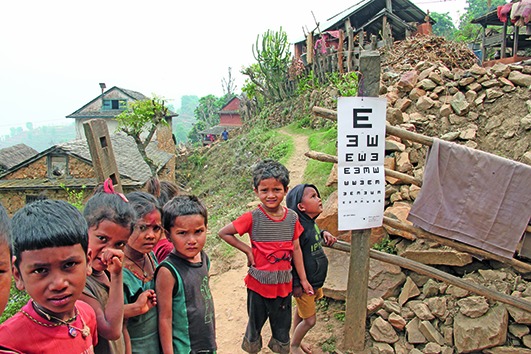
IAPB Photo competition/Manish Kumar

## Medical supplies to Yemen

WHO airlifted over 500 tons of essential medicines and medical supplies to Yemen in August through several shipments.

These shipments contained: medicines to cover almost 50% of the needs of cancer patients for one year; 100 nutrition kits to prevent severe acute malnutrition in 5000 children; medicines for other non-communicable diseases; and medical equipment needed by health facilities across the country.

“This prolonged war has caused many of the acute health needs to go unmet, due to severe shortages of life-saving medical supplies. Therefore, the arrival of these life-saving cargos has been critical to the response.” said Nevio Zagaria, WHO Representative to Yemen.

In Yemen, only 50% of the total health facilities are fully functioning, and these health facilities face severe shortages in medicines, equipment, and staff, due not only to difficulties in importing medicines and critical supplies, but also to a lack of operational funds.

http://bit.ly/2CIdLzR

## Trauma needs in Libya

As of 3 September, violence in southern Tripoli, Libya has left almost 50 people dead and more than 125 others injured and in need of urgent, life-saving medical care. The situation remains volatile. 

WHO, in collaboration with the Ministry of Health, deployed 10 mobile emergency trauma units last month consisting of doctors, paramedics, essential medicines and equipment to areas where fighting is ongoing. 

WHO has also delivered trauma medicines for 200 critical cases, and has enough medicines for 2000 more cases on standby to deliver to hospitals as needed. 

Medicines for the treatment of chronic disease have been delivered to health facilities in areas hosting people who have been displaced as a result of the violence. WHO teams also visited schools and other locations where displaced people are seeking shelter.

Attacks on health care facilities have also been reported, with a physiotherapy centre in Ain Zara partially damaged by a shell on 2 September. “With greater numbers of injured civilians expected, it is imperative that doctors and other health staff be allowed to move freely, so they can save lives without delay, and without risk to their own personal safety.” said Syed Jaffar Hussain, WHO Representative in Libya.

http://bit.ly/2xcVsx5

## Funding shortage 

WHO is appealing for US$ 11 million to provide life-saving health care to people in the Syrian governorates of Aleppo, Hama, Idlib and Lattakia. 

Hundreds of thousands of people, many of whom have been previously displaced, may be displaced again, as they flee growing insecurity and violence. 

The situation in Idlib is particularly dire; more than half a million people have been displaced to and within the governorate since January 2017.

Many internally-displaced people are living in makeshift, overcrowded shelters with little access to health care and safe water and sanitation. Poor health following years of conflict makes them vulnerable to communicable diseases. 

Rates of acute malnutrition are likely to increase. Moreover, a decline in vaccination coverage rates may lead to renewed outbreaks of vaccine-preventable diseases such as polio, jeopardizing efforts to eradicate the disease worldwide.

The humanitarian community is finding itself increasingly compromised as a large funding deficit for health has placed millions of vulnerable Syrians at risk.

“The health situation in northwest Syrian Arab Republic is already dire and looks set to deteriorate. If WHO does not receive additional funding, more than two million people caught in the cross-fire may have no access to essential health care services, including life-saving trauma care,” said Michel Thieren, WHO Regional Emergencies Director. 

WHO will use any additional funds received from donors to support primary health care, childhood vaccination and trauma services in northwest Syrian Arab Republic. WHO will also strengthen referral systems to ensure that critically ill and wounded patients can be transferred to hospitals for specialized care, as well as facilitate medical evacuations and deliver essential medicines and equipment to hospitals, clinics and mobile teams.

**Looking ahead:**

**9 October – WHO 2018 symposium on health financing for UHC, online**

**15 - 17 October – Transforming Nutrition Science for Better Health, London, The United Kingdom of Great Britain and Northern Ireland**

**16-17 October – Third Global Conference on Health and Climate Change, St. George’s, Grenada**

**21-27 October – International lead poisoning prevention week of action**

**25-27 October – Global Conference on Primary Health Care, Astana, Kazakhstan **

**30 October - 1 November – WHO’s First Global Conference on Air Pollution, Geneva, Switzerland**

